# Association between Lower Normal Free Thyroxine Concentrations and Obesity Phenotype in Healthy Euthyroid Subjects

**DOI:** 10.1155/2014/104318

**Published:** 2014-04-28

**Authors:** Jeong Ah Shin, Eun young Mo, Eun Sook Kim, Sung Dae Moon, Je Ho Han

**Affiliations:** ^1^Division of Endocrinology and Metabolism, Department of Internal Medicine, College of Medicine, The Catholic University of Korea, 222 Banpo-daero, Seocho-gu, Seoul 137-701, Republic of Korea; ^2^The Catholic University of Korea Incheon St. Mary's Hospital, 665 Bupyung-6-dong, Bupyung-gu, Incheon 403-720, Republic of Korea

## Abstract

We investigated whether thyroid function could identify obesity phenotype in euthyroid subjects. A cross-sectional analysis was performed among nondiabetic, euthyroid subjects. We stratified subjects into four groups by BMI and insulin resistance (IR). Of 6241 subjects, 33.8% were overweight or obese (OW/OB) and 66.2% were normal weight (NW). Free thyroxine (FT4) levels were negatively associated with body mass index, waist circumference, triglyceride, c-reactive protein, and HOMA-IR and positively with high-density lipoprotein cholesterol in both genders. In multivariate regression analysis, FT4 level, a continuous measurement, was negatively correlated with HOMA-IR (*β* = −0.155, *P* < 0.001 in men; *β* = −0.175, *P* < 0.001 in women). After adjustment for age, sex, metabolic, and life style factors, subjects in the lowest FT4 quartile had an odds ratio (OR) for IR of 1.99 (95% confidence interval 1.61–2.46), as compared to those in the highest quartile. The association between low FT4 and IR remained significant in both NW and OW/OB subgroups. In conclusion, low normal FT4 levels were independently related to IR in NW and OW/OB euthyroid subjects. Further studies are needed to investigate the mechanisms by which low FT4 levels are linked to high IR in euthyroid ranges.

## 1. Introduction

Obesity is closely associated with increased risk of cardiovascular disease (CVD) and is related to insulin resistance and its associated abnormalities such as hyperglycemia, hyperlipidemia, and hypertension [1]. However, obesity is not necessarily synonymous with insulin resistance, despite their strong correlation [2]. Current data describe that obese (OB) and normal weight (NM) subjects could be divided into “metabolically healthy” and “metabolically unhealthy” subsets according to insulin resistance or its related factors, suggesting that these distinctions are better predictors for the development of CVD and increased mortality than obesity itself [3]. However, related factors and underlying mechanisms for these paradoxical associations between obesity and insulin sensitivity or NW and insulin resistance have not been clarified.

Thyroid hormone regulates basal energy expenditure and influences the glucose and lipid metabolism [4]. Epidemiological studies have demonstrated increased CV events and mortality in subjects with subclinical as well as overt hypothyroidism, proposing substantial impact of thyroid insufficiency on atherosclerotic vascular changes in a graded manner [5]. This is further supported by close associations between thyroid insufficiency and clusters of metabolic abnormalities such as obesity and lipid profiles, observed even within physiological ranges [6–10], suggesting insulin resistance as a potential mechanistic link to the promotion of atherosclerosis. However, a limited number of data exist on the association between thyroid function and insulin activity in euthyroid subjects, especially when considering the influence of obesity.

Therefore, in this study we explored the relationship between thyroid function measured by FT4 and TSH levels and obesity and insulin resistance in healthy euthyroid subjects. Further, we investigated whether thyroid function could be an indicator of an insulin resistant phenotype in OB and normal NW subjects.

## 2. Methods

### 2.1. Study Population

A cross-sectional analysis was conducted among healthy, nondiabetic Koreans who underwent a general health check-up at Seoul St. Mary's Hospital Health Promotion Center between March 2009 and December 2010. A total of 8701 subjects between the ages of 20 and 80 years with data on a thyroid function and fasting plasma insulin (FPI) concentrations were considered for the present study. Exclusion criteria were: (i) TSH values out of the normal range, (ii) use of antithyroid drugs or thyroid hormone medication, (iii) a history of diabetes (or fasting serum glucose ≥ 126 mg/dL), cardiovascular, cerebrovascular, or peripheral vascular disease, (iv) serum creatinine levels ≥ 1.5 mg/dL, (v) body mass index (BMI) < 18.5 kg/m^2^: 6241 subjects were included in the final analysis. This study was approved by the institutional review board of Catholic University College of Medicine, Seoul, Korea. The informed consent was not required due to the retrospective nature of our study.

### 2.2. Measurement of Anthropometric and Biochemical Parameters

Medical history and social-behavioral information were collected through questionnaires completed by patients. Physical examinations were performed by measuring height, weight, waist circumference (WC), and blood pressure (BP) according to standardized methods. During measurements, the subjects were barefoot and wearing light clothing. BMI was calculated by dividing weight by the height squared (kg/m^2^). Before the measurement of BP, the subjects were resting in a sitting position for 10 minutes. BP was measured twice with at least a 5 min interval to obtain an average value. Blood samples were collected after the subjects had fasted at least 10 hours. The fasting plasma glucose (FPG), hemoglobin A1c (HbA1c), total cholesterol (TC), triglyceride (TG), high-density lipoprotein cholesterol (HDL-C), and low-density lipoprotein cholesterol (LDL-C) were determined enzymatically using the Hitachi 7600 chemistry analyzer (Hitachi, Tokyo, Japan). The serum CRP level was measured with a turbidimetric immunoassay (Wako Chemicals GmbH, Neuss, Germany). Fasting insulin levels were measured using commercially available RIA kits (Insulin RIA beads, TFB-Japan Co. Ltd., Japan). The serum FT4 and TSH levels were measured by enzyme immunoassay using a commercially available kit (ADVIA Centaur, Seimens, Germany). The reference ranges of FT4 and TSH were 0.93–1.70 ng/dL and 0.35–5.50 mU/L, respectively.

### 2.3. Definition of Metabolic Abnormalities and Insulin Resistance

Subjects were classified as being normal weight (NW: BMI, 18.5–24.9 kg/m^2^), overweight (OW: BMI, 25.0–29.9 kg/m^2^), or obese (OB; BMI, ≥ 30.0 kg/m^2^). Hypertension was defined as systolic blood pressure (SBP) ≥ 140 mm Hg or diastolic blood pressure (DBP) ≥ 90 mm Hg or the use of antihypertensive drugs whereas hyperlipidemia was defined as total cholesterol ≥ 240 mg/dL, TG ≥ 150 mg/dL or the use of antihyperlipidemic drugs. The degree of insulin resistance assessed by homeostasis model assessment of insulin resistance (HOMA-IR) calculated as follows: HOMA-IR = fasting insulin (*μ*U/mL) × FPG (mmol/L)/22.5. Subjects were classified as being insulin resistant (top quartile of the HOMA-IR distribution) or insulin sensitive (lower 3 quartiles of HOMA-IR distribution).

### 2.4. Statistical Analysis

Statistical analyses were performed using SAS version 9.1 (SAS Institute, Cary, NC, USA). Data were expressed as mean ± SD or number (percentage) unless otherwise stated. A one-way analysis of variance for continuous variables and the chi-square test for categorical variables were performed to compare characteristics of the study population. Pearson's correlation analyses were performed to examine the association between FT4 and TSH levels with various parameters. Due to skewed distribution, log-transformed FT4, TSH, TG, FPI, HOMA-IR, and CRP were used in the analysis including them as a continuous variable.

To determine the independent association between FT4 concentrations and insulin resistance as a continuous measure, multiple linear regression analysis was performed. FT4 quartiles were categorized separately as follows: Q1, < 1.22; Q2, 1.22–1.33; Q3, 1.34–1.48; and Q4, ≥ 1.49 mg/dL for men; Q1, < 1.13; Q2, 1.13–1.25; Q3, 1.26–1.40; and Q4, ≥ 1.41 mg/dL for women. The odds ratios (ORs) and 95% confidence intervals (CI) for insulin resistance were assessed using multivariate logistic regression analyses according to quartile of FT4 levels.* P* values of < 0.05 were considered statistically significant.

## 3. Results

### 3.1. Characteristics of Subjects Categorized by Obesity and Insulin Resistance

Of 6241 subjects, 33.8% were OW/OB and 66.2% were NW. In the subgroup of the highest quartile of insulin resistance, 1145 were OW/OB-IR and 965 were NW-IR (Table 1). Individuals in the OB/OW category were more likely to be male and to have unfavorable metabolic profiles such as higher BMI, WC, SBP, DBP, TC, TG, HDL-C, FPI, and HOMA-IR, whereas they were less likely to do regular exercise, as compared to those in the NW category. Similarly, individuals in the insulin-resistant groups had higher adiposity, SBP, DBP, TG, HDL-C, FPG, FPI, and HOMA-IR and they showed lower FT4 and HDL-C, as compared to subjects in the insulin sensitive groups within the same BMI category. In contrast, subjects in the OW/OB-IS group showed similar levels of SBP, DBP, TG, and CRP compared with those in the NW-IR group, but lower levels of FPG, FPI, and HOMA-IR.

### 3.2. Associations between Anthropometric and Metabolic Parameters and Thyroid Hormone Concentrations, as Continuous and Categorized Forms

**Figure 1:**
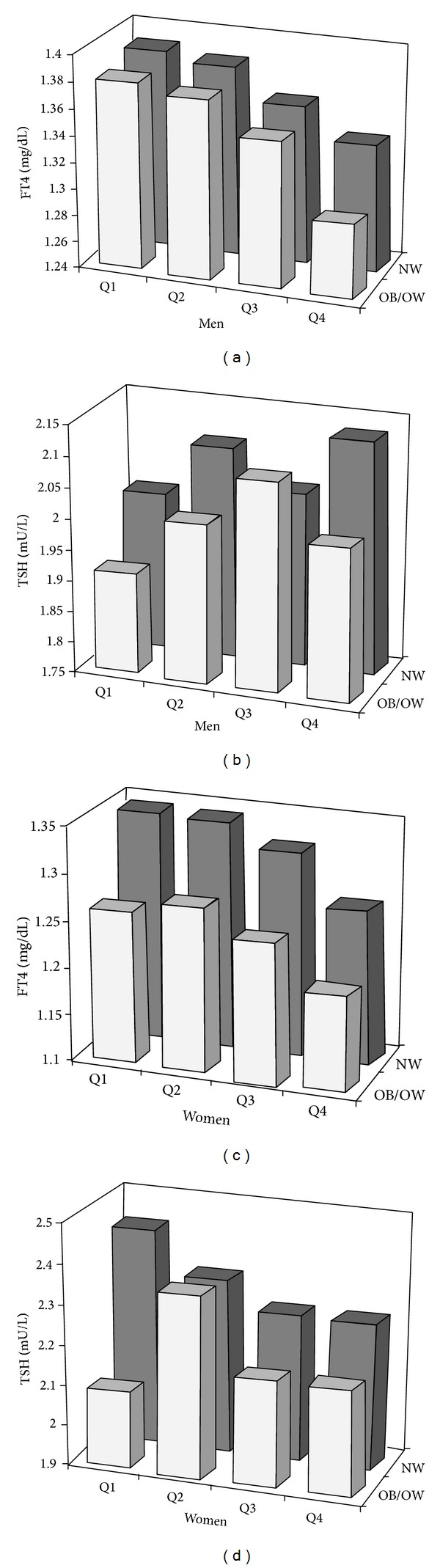
Age-adjusted relationship between HOMA-IR quartiles, obesity categories, and FT4 or TSH in men (a, b) and women (c, d).

FT4 levels were negatively associated with cardiometabolic risk factors such as BMI, WC, TG, FPI, CRP, and HOMA-IR, and positively with HDL-C, but no differences were observed in FPG and BP levels in both men and women. TSH levels showed a rather weak association with BMI and HOMA-IR only in women (Table 2). As shown in Figure 1, the FT4 level decreased in accordance with the increasing quartiles of HOMA-IR and higher degree of obesity in men and women (all *P* < 0.01). However, no differences in TSH existed across HOMA-IR quartiles by obesity groups except a very week association in NW women (*P* = 0.046). In a multivariate linear regression model including obesity measures and insulin resistance together, HOMA-IR were significantly associated with FT4 levels both in men (*β* = −0.155, *P* < 0.001) women (*β* = −0.175, *P* < 0.001) (Table 3). On the other hand, there is no association between TSH levels and HOMA-IR in both genders. Furthermore, FT4 levels were significantly associated with HOMA-IR (*β* = −0.126, *P* < 0.001) using a multivariate linear regression model adjusted for age, sex, BMI, WC, hypertension, heart rate, hyperlipidemia, and TSH (see Supplementary Table 1 in Supplementary Material available online at http://dx.doi.org/10.1155/2014/104318).

### 3.3. Risk for Insulin Resistance according to Serum FT4 Quartiles

The prevalence of insulin resistance increased significantly with decreasing FT4 quartiles for the total and each subgroup divided by obesity (Table 4). Multivariate logistic analysis was performed to investigate an independent association between FT4 levels and insulin resistance. After adjusting for age, sex, WC, heart rate, hypertension, hyperlipidemia, regular exercise, and smoking, ORs (95% CIs) for having insulin resistance from the highest to lowest FT4 quartiles, Q4 to Q1, were 1 (Ref), 1.26 (1.01–1.57), 1.54 (1.24–1.93), and 1.99 (1.61–2.46) for the entire group (*P* for trend < 0.001; Model 3). In the subgroup analysis, the fully adjusted ORs (95% CIs) for the lowest versus the highest quartile were 1.76 (1.31–2.38) in NW and 2.19 (1.62–2.96) in OW/OB groups, respectively. Further adjustment for log-transformed TSH levels did not change any of the associations presented in Table 4.

## 4. Discussion

Several studies have highlighted subsets of obesity, such as metabolically healthy but obese or metabolically unhealthy but normal weighted subjects. Previous epidemiological studies have suggested that this concept could improve risk prediction of CV events, as compared to using risk assessment based on BMI alone [3]. A recent meta-analysis revealed that an unhealthy metabolic status provides an increased risk for all-cause mortality and CV events regardless of normal weight, overweight, or obesity. Those analyses stressed the importance to assess the metabolic status in addition to BMI in the evaluation of long-term outcomes [11]. However, the underlying mechanisms that provoke insulin resistance in lean subjects or protect against it in obese subjects remain unclear. Clinical studies have reported that visceral fat [11–14], older age, physical inactivity, and smoking [15, 16] are associated with the “at risk” phenotype. In addition, close relation to biochemical markers including lipid profiles [14], hepatic enzymes [11, 12, 17], adipokines [18, 19], and IGF-1 [20] suggests that these factors could contribute to obesity phenotypes.

In the present study, we found that small differences in FT4 levels were closely associated with obesity, lipid profiles, and insulin resistance in euthyroid healthy subjects. In addition, lower FT4 activity within reference ranges was significantly associated with insulin resistance after adjusting for central obesity and other confounding factors. The association between low normal FT4 levels and insulin resistance remained significant even in the subgroups divided into NW and OB/OW subjects, suggesting that thyroid activity may be involved in the development of obesity subtypes.

Thyroid hormone regulates the basal energy expenditure and influences glucose and lipid metabolism [4]. Accordingly, a number of studies have reported associations between thyroid function and obesity and obesity-related conditions such as hyperglycemia, hypertension, hyperlipidemia, and metabolic syndrome [6–10]. In the present study, we found inverse associations between FT4 levels within the reference ranges and adiposity quantified by BMI and WC, consistent with previous studies [21–23]. Several plausible mechanisms have been postulated. One explanation is that reduced thyroid function causes obesity through a lower basal metabolic rate, in line with the mechanisms suggested in hypothyroidism [4]. This is further supported by another study, which found that slightly decreased thyroid function within the normal range was correlated with weight gain during a 5-year follow-up in 4082 subjects [24]. Another explanation is that thyroid hormone levels could be altered as a consequence of obesity. Several studies have suggested a role for obesity in the development of thyroid insufficiency in the tissue based on reduced expression of T3 and TSH receptors in the fat of obese subjects [25]. Other researchers have interpreted the frequent observation of isolated TSH elevation in obesity as a mere manifestation rather than functional defect, resulting from deranged hypothalamic-pituitary axis or effects of increased leptin on TRH production and type 2 iodothyronine deiodinase (D2) inhibition in the thyrotrophs [26]. As obese individuals frequently show high insulin resistance, discerning an independent association between insulin resistance, obesity, and thyroid function is difficult. A few studies have investigated the associations between thyroid function and insulin resistance considering the impact of obesity in euthyroid ranges, showing significant relationships with mild thyroid dysfunction [10, 27, 28], although this is controversial [22, 29, 30]. Notably, the current study identified a clear association between thyroid hormone levels and HOMA-IR, independent of adiposity, which remained statistically significant after adjustment for obesity measures or by subgroup analysis including NW subjects, free from any confounding effect of obesity. This finding is important due to the growing evidence indicating that a slight change in thyroid function in euthyroid subjects is significantly associated with atherosclerotic vascular changes (as shown in our previous data) [31]. Therefore, thyroid function may play a role in the pathogenesis of atherosclerosis via increased insulin resistance even in euthyroid ranges. However, higher insulin resistance has been reported in both hyperthyroidism and hypothyroidism, as compared to euthyroidism [32]. These contrasting findings may be explained by the divergent effects of thyroid hormone on different tissues. In hyperthyroidism, thyroid hormone can antagonize insulin action on the liver leading to increased hepatic glucose output by the sympathetic pathway from the hypothalamus and transcriptional regulation of metabolic genes [32]. Conversely, peripheral insulin resistance in muscle and adipose tissue could develop via a link to low leptin levels, reduced muscle oxidative capacity, impaired GLUT4 expression, and diminished blood flow in hypothyroidism [33, 34]. Although the physiological mechanism could not be determined in the current study, the graded association between FT4 levels and insulin sensitivity in euthyroid subjects postulates the possible influence of low FT4 levels on insulin resistance by mechanisms related to hypothyroidism to a lesser degree. Additionally, hyperinsulinemia could promote thyroid insufficiency through increased D2 activity in the thyrotrophs [35]. However, compared to somewhat weak and undetermined biological relationships, in the present study we found strong associations between FT4 levels and HOMA-IR, independent of central adiposity. Thus, as suggested by previous studies, this association might be explained by an indirect link via shared genetic [36] or environmental factors [37] influencing both thyroid function and insulin resistance in euthyroid subjects rather than a directly linked casual relation. For example, insulin resistance has been reported in subjects with D2 Thr92Ala [38] and D2 knockout mice regardless of weight gain [39].

Interestingly, the current study demonstrates that lower FT4 levels, rather than higher TSH levels, are associated with insulin resistance. Our data is consistent with other studies that found significant association between FT4, but not TSH, with various clinical outcomes such as metabolic syndrome [10], hepatic steatosis [40], bone mass density [41], frailty [42], and atrial fibrillation [43]. We could not explain why such divergent associations exist between thyroid markers and clinical outcomes, but serum FT4 could be a more reliable marker of tissue thyroid status as previous studies suggest [10, 42, 43]. Although hypothalamic-pituitary-thyroid (HPT) axis has a key role in thyroid hormone homeostasis, peripheral thyroid activity ultimately depends on circulating T4 levels and local regulatory mechanisms of thyroid hormone that involve intracellular transportation, deiondination of T4 into active T3, and binding to nuclear receptors [44]. TSH has been considered as a highly sensitive measure of the thyroid dysfunction, but current issues challenge inverse log TSH-FT4 relationship and demonstrate complex nature of the TSH response to changes in FT4 [45]. In addition, population-based studies of euthyroid subjects have reported wide variations of FT4/TSH between individuals in contrast to narrow intraindividual variation of TSH, suggesting individual setpoint of the HPT axis, inherited as a genetic trait [46].

Our study gives clinical implications as the detection of low normal FT4 levels could indicate metabolically unhealthy subjects with combined risk factors who can benefit from early screening and medical intervention of combined risk factors. Further fundamental studies could confirm whether changed thyroid activity has a protective role in obese subjects against insulin resistance or low normal activity promotes it in nonobese euthyroid subjects.

There are several limitations in the present study. First, the cross-sectional study design limit confers causal influence of the relative thyroxine deficit on insulin resistance. Second, as this study included FT4 and TSH concentrations for thyroid function screening in a general population voluntarily receiving a health exam, the total T4 and total or free T3 levels were unavailable. As moderately elevated T3 levels are commonly observed in obese subjects [26], inverse associations between FT4 and obesity parameters in the present study possibly arose from increased free T3/FT4 ratio, a surrogate of high deiodinase activity (which converts T4 to T3). However, contradictory data exist on the metabolic impact of the free T3/FT4 rato. An experimental study observed that mice with a disrupted Dio2 gene (D2KO) were susceptible to diet-induced obesity and insulin-resistance independent of obesity [39] and suggested involvement of D2 activity in the development of metabolic abnormalities. Measurement of T3 levels could help in the understanding of thyroid hormone physiology by determining the association between the free T3/FT4 ratio and obesity and insulin resistance. Third, we did not measure insulin resistance using the standard method; however, HOMA-IR has been used as a tool for measuring insulin resistance in a large-scale community-based study and was well correlated with insulin resistance as measured by the hyperinsulinemic clamp method [47]. Lastly, thyroid autoimmunity was not measured, which may have provided further insights. The strengths of our study include the large healthy population surveyed and the careful measures taken to minimize confounding variables.

In conclusion, low normal FT4 levels were independently related to “metabolically unhealthy” in NW and OW/OB euthyroid subjects. Further studies are needed to investigate the mechanisms by which low FT4 levels are linked to high insulin resistance in euthyroid ranges.

## Supplementary Material

FT4 levels were significantly associated with HOMA-IR (*β* = −0.126, P < 0.001) using a multivariate linear regression model adjusted for age, sex, BMI, WC, hypertension, heart rate, hyperlipidemia, and TSH

## Figures and Tables

**Table 1:** Clinical characteristics of the 6241 subjects.

	NW-IS	NW-IR	OB/OW-IS	OB/OW-IR	*P*
*N*	3536	595	1145	965	
Male sex (%)	1649 (46.6)	279 (46.9)	809 (70.7)	667 (69.1)	<0.001
Age (years)	49.6 ± 10.9	50.0 ± 11.2	51.6 ± 10.5^a,b^	50.4 ± 10.9	<0.001
FT4 (mg/dL)*	1.33 ± 0.22	1.28 ± 0.22^a^	1.33 ± 0.22^b^	1.27 ± 0.22^a,c^	<0.001
TSH (mIU/L)*	2.20 ± 1.14	2.20 ± 1.11	2.08 ± 1.08^a^	2.04 ± 1.05^a^	0.001
BMI (kg/m^2^)	22.1 ± 1.7	22.9 ± 1.5^a^	26.8 ± 1.7^a,b^	28.6 ± 3.5^a,b,c^	<0.001
WC (cm)	81.0 ± 5.8	83.5 ± 5.7^a^	92.0 ± 6.1^a,b^	97.0 ± 9.3^a,b,c^	<0.001
SBP (mmHg)	118.5 ± 13.6	123.3 ± 14.2^a^	125.2 ± 13.2^a^	128.9 ± 13.9^a,b,c^	<0.001
DBP (mmHg)	69.5 ± 9.7	73.0 ± 9.9	73.7 ± 9.4^a^	76.2 ± 9.3^a,b,c^	<0.001
Heart rate (beat/min)	63.1 ± 9.1	66.6 ± 9.6^a^	62.9 ± 8.7^b^	66.0 ± 9.6^a,c^	<0.001
TC (mg/dL)	201.1 ± 33.9	204.0 ± 37.7	206.6 ± 34.3^a^	209.8 ± 37.3^a,b^	<0.001
TG (mg/dL)*	85.4 ± 55.0	129.2 ± 85.1^a^	119.6 ± 75.9^a^	155.4 ± 93.6^a,b,c^	<0.001
HDL-C (mg/dL)	56.4 ± 13.0	52.5 ± 12.5^a^	49.5 ± 10.8^a,b^	47.0 ± 10.2^a,b,c^	<0.001
FPG (mg/dL)	85.5 ± 10.5	95.9 ± 10.3^a^	88.1 ± 10.1^a,b^	96.4 ± 10.7^a,c^	<0.001
FPI (U/L)*	4.8 ± 2.1	12.2 ± 4.0^a^	6.1 ± 2.0^a,b^	14.9 ± 6.9^,b,c^	<0.001
HOMA-IR*	1.0 ± 0.5	2.9 ± 1.0^a^	1.3 ± 0.5^a,b^	3.5 ± 1.8^a,b,c^	<0.001
CRP (mg/dL)^∗†^	0.16 ± 0.38	0.20 ± 0.58^a^	0.16 ± 0.26^a^	0.25 ± 0.52^a,b,c^	<0.001
Hypertension (%)	536 (15.2)	149 (24.0)	309 (27.0)	333 (34.5)	<0.001
Hyperlipidemia (%)	781 (22.1)	238 (40.0)	432 (37.7)	503 (52.1)	<0.001
Current smoker (%)^‡^	636 (19.9)	103 (20.7)	272 (26.2)	199 (24.8)	<0.001
Regular exercise (%)^§^	870 (29.0)	131 (28.9)	251 (26.3)	163 (22.2)	0.002

Data are expressed as means ± SD or number (percentage) unless otherwise indicated.

NW: normal weight; OW/OB: overweight/obese; IS: insulin sensitive; IR: insulin resistance; BMI: body mass index; WC: waist circumference; WHR: waist-hip ratio; SBP: systolic blood pressure; DBP: diastolic blood pressure; TG: triglyceride; HDL-C: high-density lipoprotein cholesterol; LDL-C: low-density lipoprotein cholesterol; FPG: fasting plasma glucose; FPI: fasting plasma insulin; HOMA-IR; CRP: C-reactive protein.

One-way ANOVA and Tukey's post-hoc for continuous variables. ^a^ *P* < 0.05 versus NW-IS; ^b^ *P* < 0.05 versus NW-IR; ^c^ *P* < 0.05 versus OW/OB-IS; *tested by log-transformed; ^†^measured in 4877 subjects; ^‡^measured in 5529; ^§^regular exercise was defined as exercise for more than 30 minutes at a time more than three times a week (measured only in 5140 subjects).

**Table 2:** Age-adjusted Pearson's correlation coefficients of FT4 and TSH with clinical variables.

	FT4*		TSH*	
	Men	Women	Men	Women
Age	−0.15^‡^	−0.03	0.03	0.06^‡^
BMI (kg/m^2^)	−0.14^‡^	−0.12^‡^	−0.01	−0.05^‡^
WC (cm)	−0.14^‡^	−0.09^‡^	−0.02	−0.03
SBP (mmHg)	−0.01	−0.01	0.001	−0.02
DBP (mmHg)	0.002	−0.01	0.03	−0.01
Heart rate (beat/min)	0.06^‡^	0.07^‡^	−0.04^†^	−0.06^‡^
TC (mg/dL)	0.05^‡^	0.04	0.02	0.05^‡^
TG (mg/dL)*	−0.05^‡^	−0.14^‡^	0.08^‡^	0.04^†^
HDL-C (mg/dL)	0.09^‡^	0.09^‡^	−0.03	−0.003
FPG (mg/dL)	−0.04^†^	−0.02	0.03	0.02
FPI (U/L)*	−0.18^‡^	−0.17^‡^	0.01	−0.07^‡^
HOMA-IR*	−0.17^‡^	−0.16^‡^	0.02	−0.05^‡^
hsCRP*	−0.07^‡^	−0.06^‡^	−0.03	−0.03

*Tested by log-transformed; ^†^ *P* < 0.05; ^‡^ *P* < 0.01.

**Table 3:** Multiple regression analysis of determinants of serums FT4 and TSH concentrations.

	FT4*				TSH*			
	Men		Women		Men		Women	
	*β* ^a^	*P*	*β*	*P*	*β*	*P*	*β*	*P*
Age	−0.149	<0.001	0.025	0.233	0.032	0.083	0.062	0.004
BMI	−0.016	0.644	−0.116	0.002	−0.009	0.803	−0.071	0.059
WC	−0.064	0.063	−0.064	0.091	−0.039	0.267	0.050	0.189
Hypertension	−0.008	0.662	0.050	0.013	−0.011	0.541	−0.031	0.131
Heart rate	0.087	<0.001	0.103	<0.001	−0.050	0.004	−0.046	0.016
Hyperlipidemia	0.042	0.017	0.031	0.116	0.062	<0.001	0.028	0.154
HOMA-IR*	−0.155	<0.001	−0.175	<0.001	0.040	0.054	−0.036	0.099
*R* ^2^ (%)	6.4		4.5		0.9		1.1	

*Tested by log-transformed; ^a^standardized coefficient. These results are adjusted for all of the other variables listed in the table.

**Table 4:** Adjusted odd ratios (ORs) and 95% confidence intervals (CIs) for insulin resistance according to FT4 quartiles.

	Quartile of FT4 levels (mg/dL)				
	Q1	Q2	Q3	Q4	*P* for trend
Total					
*N*	1602	1458	1626	1555	
Model 1	2.17 (1.84–2.57)	1.62 (1.37–1.93)	1.33 (1.12–1.58)	1 (Ref)	<0.001
Model 2	1.99 (1.65–2.40)	1.54 (1.27–1.87)	1.21 (1.00–1.47)	1 (Ref)	<0.001
Model 3	1.99 (1.61–2.46)	1.54 (1.24–1.93)	1.26 (1.01–1.57)	1 (Ref)	<0.001
NW					
*N*	970	945	1110	1106	
Model 1	1.76 (1.37–2.26)	1.46 (1.13–1.89)	1.30 (1.01–1.68)	1 (Ref)	0.001
Model 2	1.85 (1.43–2.40)	1.48 (1.13–1.94)	1.21 (0.93–1.57)	1 (Ref)	<0.001
Model 3	1.76 (1.31–2.38)	1.48 (1.08–2.02)	1.27 (0.94–1.73)	1 (Ref)	0.002
OW/OB					
*N*	632	513	516	449	
Model 1	2.23 (1.74–2.87)	1.60 (1.24–2.08)	1.30 (1.00–1.69)	1 (Ref)	<0.001
Model 2	2.13 (1.62–2.79)	1.60 (1.21–2.12)	1.21 (0.92–1.61)	1 (Ref)	<0.001
Model 3	2.19 (1.62–2.96)	1.60 (1.17–2.20)	1.23 (0.90–1.68)	1 (Ref)	<0.001

Model 1: adjusted for age and sex.

Model 2: adjusted for age, sex, BMI, WC, heart rate, hypertension, and hyperlipidemia.

Model 3: adjusted for age, sex, BMI, WC, heart rate, hypertension, hyperlipidemia, regular exercise, and smoking (total, *n* = 4885; NW, *n* = 3265; OW/OB, *n* = 1620).
