# Supplementary material for: Association between Lower Normal Free Thyroxine Concentrations and Obesity Phenotype in Healthy Euthyroid Subjects
Source: Int J Endocrinol. 2014 Apr 28;2014:104318. doi: 10.1155/2014/104318 (PMC4024385; doi:10.1155/2014/104318)
Supplement: Supplementary file 1 — FT4 levels were significantly associated with HOMA-IR (β = −0.126, P < 0.001) using a multivariate linear regression model adjusted for age, sex, BMI, WC, hypertension, heart rate, hyperlipidemia, and TSH [file 104318.f1.pdf]

Supplementary table 1. . Multiple regression analysis of determinants of HOMA-IR

	HOMA-IR*					
	Model 1		Model 2		Model 3	
	β[a]	*P*	*β*[a]	*P*	*β*[a]	*P*
Age	-0.090	< 0.001	-0.083	< 0.001	-0.090	< 0.001
Sex	0.054	< 0.001	0.083	< 0.001	0.054	< 0.001
BMI	0.289	< 0.001	0.302	< 0.001	0.289	< 0.001
WC	0.174	< 0.001	0.177	< 0.001	0.174	< 0.001
Hypertension	0.066	< 0.001	0.064	< 0.001	0.066	< 0.001
Heart rate	0.144	< 0.001	0.136	< 0.001	0.144	< 0.001
Hyperlipidemia	0.142	< 0.001	0.138	< 0.001	0.141	< 0.001
FT4*	-0.126	< 0.001	–	–	-0.126	< 0.001
TSH*	–	–	0.003	0.835	0.004	0.693
R^2 (%)	30.6		29.1		30.6	

*Tested by log-transformed; [a]Standardized coefficient. These results are adjusted for all of the other variables listed in the table.
